# The structure of a furin-antibody complex explains non-competitive inhibition by steric exclusion of substrate conformers

**DOI:** 10.1038/srep34303

**Published:** 2016-09-27

**Authors:** Sven O. Dahms, John W. M. Creemers, Yvonne Schaub, Gleb P. Bourenkov, Thomas Zögg, Hans Brandstetter, Manuel E. Than

**Affiliations:** 1 Protein Crystallography Group, Leibniz Institute on Aging-Fritz Lipmann Institute (FLI), Beutenbergstr. 11, 07745 Jena, Germany; 2 Department of Molecular Biology, University of Salzburg, Billrothstrasse 11, A-5020 Salzburg, Austria; 3 Department of Human Genetics, KU Leuven, Herestraat 49, B-3000 Leuven, Belgium; 4 European Molecular Biology Laboratory, Hamburg, Germany

## Abstract

Proprotein Convertases (PCs) represent highly selective serine proteases that activate their substrates upon proteolytic cleavage. Their inhibition is a promising strategy for the treatment of cancer and infectious diseases. Inhibitory camelid antibodies were developed, targeting the prototypical PC furin. Kinetic analyses of them revealed an enigmatic non-competitive mechanism, affecting the inhibition of large proprotein-like but not small peptidic substrates. Here we present the crystal structures of furin in complex with the antibody Nb14 and of free Nb14 at resolutions of 2.0 Å and 2.3 Å, respectively. Nb14 binds at a site distant to the substrate binding pocket to the P-domain of furin. Interestingly, no major conformational changes were observed upon complex formation, neither for the protease nor for the antibody. Inhibition of furin by Nb14 is instead explained by steric exclusion of specific substrate conformers, explaining why Nb14 inhibits the processing of bulky protein substrates but not of small peptide substrates. This mode of action was further supported by modelling studies with the ternary factor X-furin-antibody complex and a mutation that disrupted the interaction interface between furin and the antibody. The observed binding mode of Nb14 suggests a novel approach for the development of highly specific antibody-based proprotein convertase inhibitors.

Furin^1^ belongs to the family of the calcium-dependent proprotein convertases (PCs). These endoproteinases share structural homology of their catalytic domains with subtilisin. However, in contrast to subtilisin they are highly specific enzymes, activating a large number of secreted and membrane-associated secretory proteins by limited proteolysis. Substrate proteins include blood coagulation factors, hormones, growth factors, matrix metalloproteases as well as viral capsid proteins and bacterial toxins^1^,^2^. The classical PCs cleave after basic residue motifs, with furin preferentially recognising the motif R-X-K/R-R↓ (where “↓” represents the scissile peptide bond)^3^,^4^. Besides the subtilisin-like catalytic domain, all PCs require the so-called proprotein convertase domain (P-domain, for some family members also called Homo B domain) for catalytic activity^5^. The P-domain is located C-terminal to the catalytic domain and adopts a β-barrel-like fold. These proteases are also involved in a large number of pathologies, including bacterial and viral infections as well as cancer progression and metastasis^2^. Consequently, inhibitors of furin and other PCs are promising drug candidates^6^ and inhibitory molecules of a different chemical nature are currently being investigated in various labs^7^. Inhibitors of furin were successfully applied to inhibit the cell motility and invasiveness of cancer cells^8^, to impair carcinoma cell growth^9^ and to inhibit activation of HIV-1 glycoprotein gp160^10^. However, although inhibitors with high affinity have been developed^11^, obtaining specificity between the PC-family members remains challenging^12^. Most inhibitors target the substrate binding cleft of the PCs, which is highly conserved between these proteases^13^,^14^.

Crystal structures of inhibitor-bound murine^4^ and human^11^,^15^ furin as well as of the yeast homolog Kex2p^16^,^17^ and modelling approaches^13^,^14^ gave hints as to how substrates and substrate-derived inhibitors bind to the PCs. The commonly accepted notion is that the minimal working unit of the PCs consists of two consecutive structural units, the catalytic domain and the P-domain. PCs bind their cognate substrates and inhibitors *via* recognition at several subsites at the catalytic domain, typically involving multiple tight contacts and hydrogen bonds. The P-domain is hereby closely associated with the catalytic domain and is essential for its stabilisation, but it does not seem to be involved in the ultimate subsite recognition. Antibodies can act as highly specific protease inhibitors^18^. Camelid antibodies (or their minimal active subfragments, the variable heavy chain (V_H_) domains; as isolated proteins often called V_H_H-fragments or “nanobodies”) as well as antigen binding fragments (F_ab_s) have been successfully applied to effectively inhibit pharmacological targets like human growth factor activator (HGFA)^19^, matriptase^20^, tumour necrosis factor-α-converting enzyme (TACE)^21^ or the trypsin-like serine protease HtrA-1^22^. Nanobodies are highly versatile tools for research, diagnostic and therapeutic applications. They can be easily manipulated to change their half-life or to link them to another polypeptide like a toxin, a reporter, or a peptide inhibitor^23^. Structural analyses showed that antibody binding often blocks the active site cleft of target proteases (e.g. refs 19 and 20) or induces conformational changes and thus inhibits proteolysis allosterically (e.g. refs 24, 25, 26). Recently, nanobodies were developed which specifically target human and mouse furin^27^. They inhibited cleavage of diphtheria toxin and effectively protected cells from diphtheria-toxin-induced cytotoxicity. Interestingly, only the turnover of protein substrates by furin was inhibited, whereas the hydrolysis of short fluorogenic peptides remained unaltered. As the kinetic analysis revealed a non-competitive mode of inhibition, it was concluded that these nanobodies do not seem to directly interfere with the catalytic mechanism or binding of substrates to the active site cleft^27^, calling for a structural characterization of their rather unusual mode of inhibition.

To unravel the binding epitope and the mode-of-action of the furin-inhibiting nanobody Nb14 we solved its structure in isolation as well as in complex with the target protease. Our structural and biochemical data explain its unusual inhibitory properties and suggest strategies for the future of inhibitor development.

## Results

### Co-crystal structure of furin and Nb14

**Figure 1:**
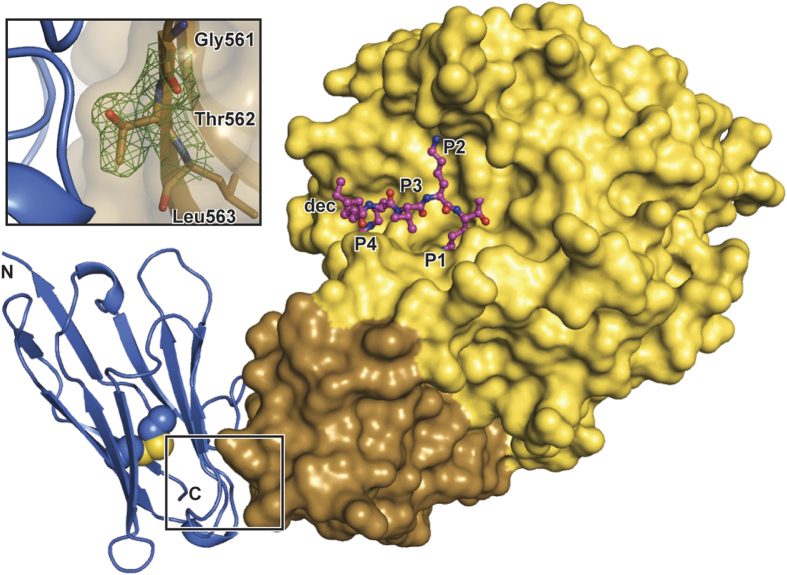
Structure of the furin:RVKR-CMK:Nb14 complex. Human furin is shown as surface representation. The catalytic domain and the P-domain are coloured in yellow and brown, respectively. The dec-RVKR-CMK inhibitor is shown as a ball and stick model with magenta carbons. Nb14 is displayed as a cartoon representation (blue) with the central disulphide bond highlighted as spheres. The inset shows a part of the interaction interface of furin and Nb14. The surface of the P-domain is shown as a partially transparent surface. The peptide stretch Gly561-Thr562-Leu563 is shown as a stick model. The 2***F***_o_−***F***_c_ electron density composite omit map (green mesh) is contoured at 1.0 σ.

We have crystallised the ternary complex of human furin with decanoyl-RVKR-chloromethylketone (RVKR-CMK) and the inhibitory camelid antibody Nb14. Even optimised crystals grew as needle clusters. Using a whole needle-cluster, initial diffraction data were obtained to a limiting resolution of 2.9 Å at the synchrotron beamline BL14.1 of the Helmholtz-Zentrum Berlin (HZB) (Fig. S1a, Table S1). These data verified the presence of well diffracting mono-crystalline fragments within the mounted crystal bundles. The structure was solved by molecular replacement revealing the presence of two copies of the furin:RVKR-CMK:Nb14 complex in the asymmetric unit. To obtain better defined crystallographic data, rather small crystal fragments with approx. sizes of only 5 × 5 × 150 μm^3^ were separated from these needle clusters and were used for single crystal diffraction data collection at the micro-focus beamline P14 at the European Molecular Biology Laboratory (EMBL) in Hamburg (Fig. S1b, Table S1). The latter dataset was subsequently used for refinement of the furin:RVKR-CMK:Nb14 complex at 2.0 Å resolution at high quality with final R/Rfree factors of 16.3/19.7% (Fig. 1, Table S1).

Nb14 was bound to the P-domain of furin rather than to the catalytic domain (Figs 1 and S2), which in addition was covalently inhibited by RVKR-CMK. The interaction interface at the P-domain is located distant to the active site cleft of the protease, involving amino acids Pro458-Asp460, Thr492-Asn496, Ala525-Asn529 and Asn558-Thr564 of furin. The complementary region at the surface of Nb14 involves the amino acid segments Tyr32-Tyr35, Trp47-Arg53, Ser56-Asp59, Val98-Ala102 as well as Trp106. Assembly analysis with the PISA-server^28^ revealed an average covered surface area of ~685 A^2^. The interaction interface (~700 A^2^) is somewhat smaller than for the HGFA-inhibiting F_ab_s Ab58 (~900 A^2^) and Ab75 (~1000 A^2^). However, it corresponds well to the average size observed for protein-antigen complexes (varying from ~400 A^2^-~1000 A^2 ^29) and to canonical kunitz-type trypsin inhibitors like amyloid precursor protein protease inhibitor domain (APPI or KPI, ~700 A^2^) and basic pancreatic trypsin inhibitor (BPTI, ~800 A^2^)^30^. The calculated average solvation free energy gain P-value (Δ^i^G-P) and complexation significance score (CSS) values of 0.199 and 0.293 of this interface underline the significance of the observed interaction. All other molecular interactions, either mediated by non-crystallographic or crystallographic contacts, are classified as non-significant. An unusually high number of tyrosine side chains is found in the interaction interface of furin and Nb14, including Tyr32, Tyr35, Tyr109 of Nb14 and Tyr560 of furin. These tyrosines form specific hydrogen bonds and contribute to the hydrophobic character of the surface. Analysis with the PISA server^28^ revealed an average solvation free energy gain (Δ^i^G) of −28.5 kJ/mol, indicating a highly hydrophobic interface.

### Mutation of the interaction interface abrogates inhibition by Nb14

To analyse the impact of the observed molecular contact, a point mutation was introduced to furin changing Thr562 into arginine. Thr562 is located at a central position of the interaction interface (inset in Fig. 1) Substitution by a bulky arginine sidechain, however, is expected to clash with the complementary surface of Nb14 and to disrupt its interaction with furin. The conservation of this amino acid in evolution is very low and its substitution should therefore not affect proteolysis^13^. Indeed, we measured very similar specific activities of 53.3 ± 1.2 μmol 7-Amino-4-methylcoumarin (AMC)/h/mg and 54.3 ± 0.5 μmol AMC/h/mg for purified wild type (Furin^WT^) and mutated human furin (Furin^T562R^), respectively.

**Figure 2:**
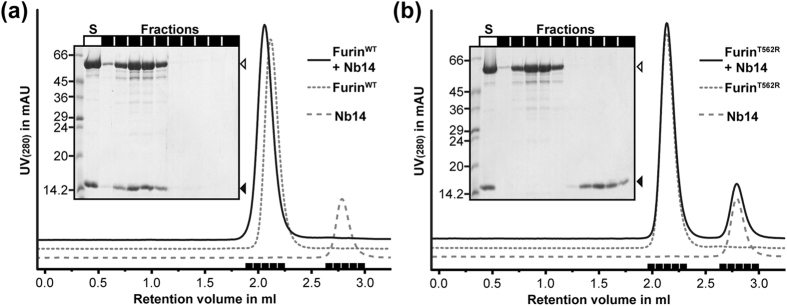
Interaction of Nb14 with Furin^WT^ or Furin^T562R^. The UV_280_ absorption is shown as a function of the elution volume of the GPC column. Fractions under the peaks (black bars) were analysed by SDS-PAGE (insets). The premixed GPC sample was loaded as control to the SDS-PAGE (S). (**a**) GPC run of Nb14 and Furin^WT^, marked in the inset as open and filled arrowheads, respectively. (**b**) GPC run of Nb14 and Furin^T562R^, marked in the inset as open and filled arrowheads, respectively.

Next, we analysed the binding properties of Furin^WT^ and Furin^T562R^ in analytical gel permeation chromatography (GPC) experiments. If an equimolar mixture of Furin^WT^ and Nb14 is subjected to gel permeation chromatography, the proteins form the expected complex and co-elute in a single peak (Fig. 2a). Pre-mixed Nb14 and mutant Furin^T562R^, however, elute as two separated peaks from the GPC-column (Fig. 2b). In conclusion, the mutation Thr562Arg at the interaction interface of furin disrupts complex formation with Nb14.

**Figure 3:**
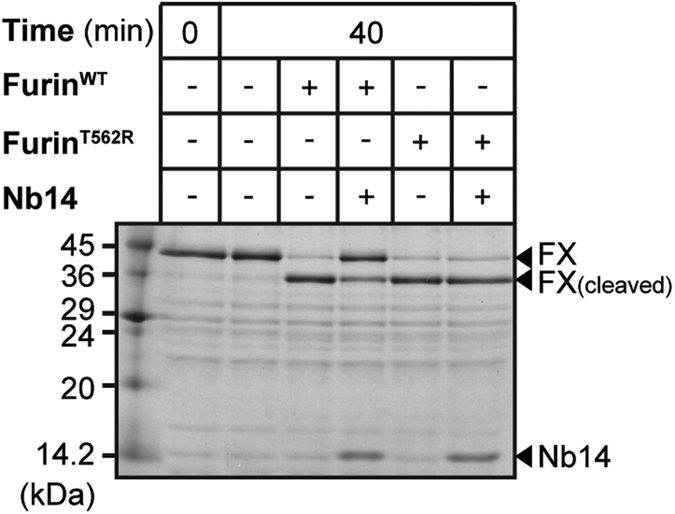
Proteolysis of factor X^S195A^ by Furin^WT^ or Furin^T562R^ and its inhibition by Nb14. Factor X^S195A^ was subjected to limited proteolysis by Furin^WT^ or Furin^T562R^ and analysed by SDS-PAGE. The bands of uncleaved factor X^S195A^ (FX), cleaved factor X^S195A^ (FX_(cleaved)_) and Nb14 are marked with arrowheads.

Inhibition of furin by Nb14 can conveniently be monitored in limited proteolysis experiments with the furin substrate coagulation factor X^31^. Maturation of it requires cleavage by furin in between the catalytic domain and the EGF-2 domain. For cleavage assays we used the inactive Ser195Ala mutant (factor X^S195A^), showing largely reduced self-degradation compared to the wild type protease^32^. After incubation of both proteins a band shift from 42 to 38 kDa is observed (Fig. 3). Furin^WT^ and Furin^T562R^ essentially showed the same activity in the factor X^S195A^ cleavage assay (Fig. 3). Apparently the introduced mutation affects neither the turnover of small peptidic substrates (see above) nor the proteolysis of protein substrates like factor X^S195A^. Cleavage of factor X^S195A^ by Furin^WT^ is largely reduced in the presence of 1 μM Nb14 indicating inhibition of furin by the antibody (Fig. 3). Furin^T562R^, however, performs very similarly with and without Nb14 in the reaction mixture (Fig. 3).

### Nb14 inhibits furin cleavage of large substrates via steric exclusion

An overall comparison of the human furin structure bound to Nb14 with isolated human furin (PDB-ID: 4RYD^11^) revealed a low average C_α_-root mean square deviation (r.m.s.d.) of 0.32 Å. Furin is found in its catalytically active state forming a typical covalent complex with the chloromethylketone compound (Fig. S3a,b) that is very similar to non-covalently inhibited human furin^15^ and covalently inhibited mouse furin^4^. To investigate the effect of furin binding on the structure of the antibody, we also solved the structure of Nb14 in its free state (Table S1, Fig. S3c, see Supplementary Information for details). Also here, no significant structural alteration could be found as also indicated by the low C_α_-r.m.s.d. of 0.20 Å (Fig. S3c).

Analysing the complex between furin and Nb14, we found that the shortest distance observed between Nb14 (Asp62, side chain carboxylate) and the decanoylamide-C9 of the inhibitor measures approx. 19 Å. Canonical binding of peptides up to the S5 or S6 pockets is thus completely unperturbed by Nb14 and the placement of an elongated peptide chain to this region is sterically not hindered. In addition, the catalytic cleft and the active site of furin are clearly not blocked by the binding of Nb14, and its inactivity towards the cleavage of small peptidic substrates^27^ is perfectly conceivable.

**Figure 4:**
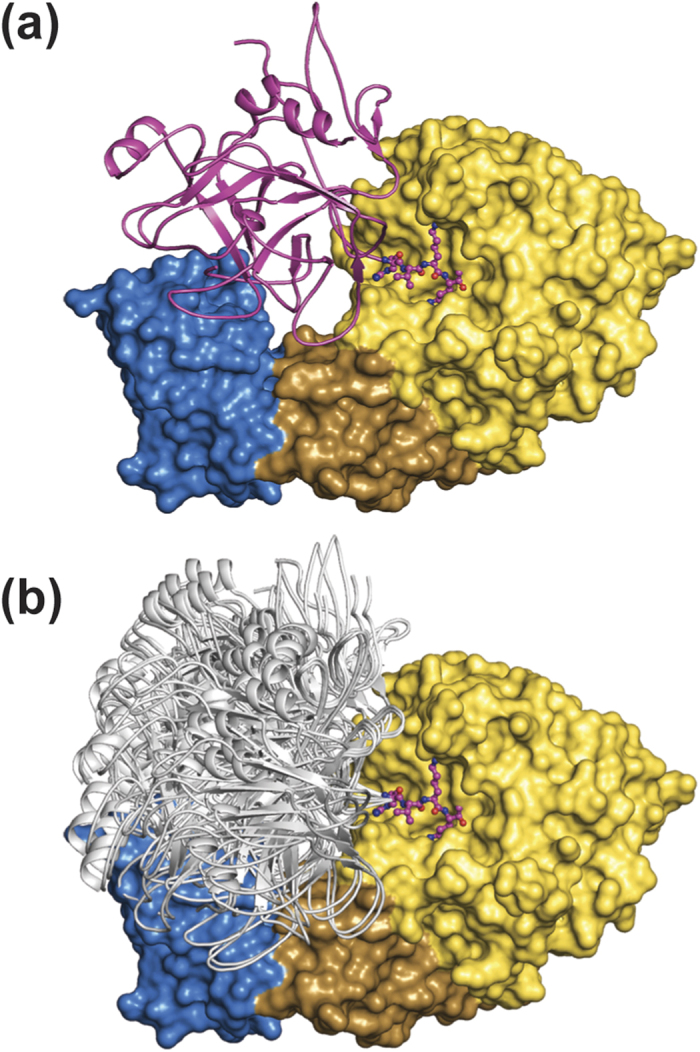
Modelling of the furin-factor X enzyme-substrate complex. The furin:Nb14 complex is shown as surface representation and coloured in yellow (furin, catalytic domain), brown (furin, P-domain) and blue (Nb14). For modelling, the catalytic domain of factor X (cartoon representation) was linked to the N-terminus of the P1-P4 tetrapeptide (stick representation, magenta) of the co-crystallised RVKR-CMK inhibitor. (**a**) The one conformer that fits to the furin:Nb14 complex is coloured in magenta. (**b**) Conformers that do not fit to the furin:Nb14 complex are shown in grey.

Globular folded parts of substrate proteins do however show an additional level of access control to the active site of proteases, as they are largely sterically restricted from the substrate binding cleft. Therefore, the binding of proteinaceous substrates to furin and their cleavage should only be possible if their overall conformation fits to the specific topology of the furin:Nb14 complex. This hypothesis was tested by means of modelling studies with the coagulation factor X (Figs 4 and S4). Proteolysis occurs here C-terminal to the recognition sequence Arg181-[P3]-Lys183-Arg184-↓. The P1-P4 amino acids are estimated to bind in a similar conformation as observed for the RVKR-CMK inhibitor complex. For our modelling studies this peptide stretch was aligned immediately C-terminal to the EGF2-domain of factor Xa (PDB-ID:2GD4^33^), creating an extended factor Xa model, Xa* (catalytic domain including the furin recognition motif). The whole catalytic domain of factor Xa* was hereby treated as a rigid body, varying initially only the relative conformation of the amino acids between Thr178 and Arg181 and later also of the peptide stretch Cys174 to Glu180. Using this approach we modelled 13 different conformers of furin-bound factor Xa* that fit to the substrate binding pocket (Figs 4 and S4a). During modelling we realised that the conformation of Xa* is largely restricted by the deep substrate binding cleft of furin and that the region between Thr178 and Arg181 requires a rather extended conformation to avoid steric clashes of furin and factor Xa. In presence of the Nb14 the conformational space is even more restricted and 12 out of the 13 initially modelled Xa* conformers now show main-chain clashes with the antibody (Figs 4 and S4b). These clashes exclusively occur at sites distant to the active site cleft of furin. Consequently, the modelling studies suggest a mode of action of Nb14 involving conformational restriction of the substrate. Binding and turnover of substrate proteins is still possible if this antibody is bound to furin, but now only a subset of all possible substrate conformers is still allowed to bind to and to be cleaved by the Nb14:furin complex.

## Discussion

We have investigated the structural basis of inhibition of human furin by the highly specific inhibitory camelid antibody Nb14^27^. The structure of the furin-nanobody complex revealed a binding epitope at the P-domain distant to the active site cleft. These data imply a unique inhibition mechanism based on steric exclusion of certain conformers during substrate binding. Using a micrometre-sized X-ray beam and a high-precision diffractometer at the PETRAIII beamline P14 was hereby essential to solve the crystallographic structure as respective complex crystals could only be obtained in the form of small needles.

The mode of action observed for Nb14 is largely different to other nanobodies that inhibit proteases by either blocking the active site cleft (e.g. refs 19 and 20) and/or by inducing conformational changes that interfere with the catalytic mechanism (e.g. refs 24, 25, 26). It also suggests how inhibitory specificity between the highly homologous PCs can be achieved. Nb14 was shown to bind exclusively to furin^27^. Other PC family members, e.g. paired basic amino acid cleaving enzyme 4 (PACE4), proprotein convertase 1 (PC1) and proprotein convertase 2 (PC2), are not recognised by Nb14^27^. Interestingly it binds to a region of furin that was previously shown to have a low degree of conservation^13^, as the surface residues of the P-domain and especially the region around Thr562 are largely variable among the PC family members (Fig. S5). Notably, a point mutation in furin at Thr562 also completely abolished binding of Nb14. In contrast, peptide-based inhibitors and related small molecules directly interact with the core of the substrate binding pocket that is much more conserved between the different family members. The specificity of such compounds for individual PC-family members is correspondingly much lower^12^.

The structural features observed for the furin:RVKR-CMK:Nb14 complex also nicely explain the reported kinetic properties of Nb14. Binding of the nanobody occurs independently of the occupation of the substrate binding cleft. In previous studies we demonstrated that covalently and non-covalently inhibited furin showed very similar structures^10^. Therefore a structural influence of the bound inhibitor on the interaction interface of Nb14 is unlikely. As observed for the RVKR-CMK peptide, small fluorogenic substrates can bind to the protease and are hydrolysed with unaltered efficacy with and without bound Nb14^27^. Proteinaceous substrates, however, occupy a large space in the non-primed region of furin’s active site cleft as shown by modelling studies with factor X. In the furin:Nb14 complex especially, this space is restricted and thus specific substrate conformers are excluded from binding, reducing the effective substrate concentration. Previous studies have shown that the inhibition strength of Nb14 indeed varied between substrates, e.g. a stronger inhibition was observed for glypican 3 (GPC3) in comparison to tumour necrosis factor β (TGFβ)^27^. These data are very much in line with the structural features of the furin:RVKR-CMK:Nb14 complex and with the modelled furin:Xa*:Nb14 enzyme substrate complex. Our data suggest that inhibition by Nb14 also seems to depend on the specific steric properties of furin’s substrates.

In conclusion, Nb14 seems to facilitate the specific inhibition of furin over other PC family members. In addition, it probably also allows some specificity for certain substrate proteins or substrate classes. This potential substrate specificity of furin inhibiting antibodies must be characterised in detail by future studies. Nonetheless, this outstanding feature of Nb14 is of great interest for pharmacological applications. Potential therapeutic side effects might be reduced by targeting the conversion of only specific substrates rather than by complete inhibition of furin or even several PCs at the same time. Therapeutic antibodies might be raised to mainly inhibit only specific substrates of furin (e.g. hemagglutinin to treat acute influenza infections). Molecular tools are available (e.g. phage display^34^) allowing protein- and epitope-specific selection of antibodies. Therefore, similar molecules like Nb14 may be developed for inhibition of other PC family members (e.g. PACE4) or other protease classes.

## Methods

The expression of camelid antibody Nb14^27^, human furin^15^ and recombinant coagulation factor X^S195A ^32 as well as the activity test of furin were described previously. Prior to crystallisation Nb14 and dec-RVKR-CMK-inhibited human furin were mixed at equimolar ratios and the resulting complex was purified by GPC. Details of the purification procedure and activity assays of the enzymes used are described in Supplementary Information.

The furin:RVKR-CMK:Nb14 complex (~9 mg/ml in in 10 mM Hepes, pH 7.5, 100 mM NaCl and 2 mM CaCl_2_) was crystallised in 0.1 M sodium acetate, pH 5.6, 16–18% PEG 3350. Crystals of isolated Nb14 were grown using a controlled dry-out procedure. Crystallisation drops (200 nl) of protein solution (10 mg/ml in 10 mM Hepes, pH 7.5, 100 mM NaCl and 1mM CaCl_2_) were pipetted without any reservoir solution. The plates were sealed and crystals appeared after several days and were stable for several weeks. Diffraction data were collected at BL 14.1 of BESSY-II, Helmholtz-Zentrum Berlin (HZB)^35^, and beamline P14, EMBL Hamburg (Table S1). Data processing was performed in XDS^36^ and programs of the CCP4-suite^37^. The structures were solved by molecular replacement in PHASER^38^ using the structures of the isolated Nb14 and human furin (PDB-ID 4RYD^11^). COOT^39^ and PHENIX^40^ were applied for model building and refinement, respectively. MAIN^41^ was used for modelling of the furin:Nb14:factor X complex. Details of data collection, structure solution, model building and refinement procedures are described in Supplementary Information.

Analytical gel permeation chromatography runs were performed on a Superdex 200 5/150 GL column (GE Healthcare) in GPC buffer (10 mM Hepes, pH 7.5, 100 mM NaCl, 1 mM CaCl_2_) using an Aekta Micro FPLC system (GE Healthcare). The proteins were premixed at an equimolar ratio or mixed with GPC buffer for control runs at final concentrations of ~23μM each and subsequently subjected to GPC at 0.2 ml/min. The peak fractions were analysed by SDS-PAGE. GPC binding assays were repeated three times.

Cleavage and inhibition assays with factor X^S195A^ were performed in 100 mM Hepes, 5 mM CaCl, 0.5% TritonX100, pH7.0 at 25 °C in 20 μl reaction setups. Samples contained 2.8 μg recombinant factor X^S195A^, 4 ng Furin^WT^ or Furin^T562R^ and 1 μM Nb14. For control samples the respective protein buffers were added to the reactions.

## Additional Information

**Accession codes:** Structure factors and coordinates of the complex structure Nb14:Furin as well as of the structure of the isolated Nb14 have been deposited in the protein databank (PDB) with accession codes 5JMO and 5JMR, respectively.

**How to cite this article**: Dahms, S. O. *et al*. The structure of a furin-antibody complex explains non-competitive inhibition by steric exclusion of substrate conformers. *Sci. Rep.* **6**, 34303; doi: 10.1038/srep34303 (2016).

## Supplementary Material

Supplementary Information
