# Supplementary material for: The structure of a furin-antibody complex explains non-competitive inhibition by steric exclusion of substrate conformers
Source: Sci Rep. 2016 Sep 27;6:34303. doi: 10.1038/srep34303 (PMC5037460; doi:10.1038/srep34303)
Supplement: Supplementary Information [file srep34303-s1.pdf]

Supplementary information

The structure of a furin-antibody complex explains non-competitive inhibition by steric exclusion of substrate conformers.

Sven O. Dahms[1, 4], John W. M. Creemers[2], Yvonne Schaub[1], Gleb P. Bourenkov[3], Thomas Zögg[4,5], Hans Brandstetter[4], Manuel E. Than[1]

[1]Protein Crystallography Group, Leibniz Institute for Age Research - Fritz Lipmann Institute (FLI), Beutenbergstr. 11, 07745 Jena, Germany

[2]Department of Human Genetics, KU Leuven, Herestraat 49, B-3000 Leuven, Belgium

[3]European Molecular Biology Laboratory, Hamburg, Germany

[4]Department of Molecular Biology, University of Salzburg, Billrothstrasse 11, A-5020 Salzburg, Austria

[5] Present address: VUB Vrije Universiteit Brussel, VIB Dept. Molecular and Cellular Interactions, Pleinlaan 2,B-1050 Brussels, Belgium

Supplementary methods

Protein expression and purification.

The camelid VHH-fragment Nb14 was expressed in the periplasm of *E. coli* as described previously [1]. The bacterial cells were centrifuged at 6000 g for 20 min and stored at -20°C. The frozen cells were resuspended carefully in lysis buffer (30 ml/1.0 OD_{600}/l culture volume; 30 mM Tris/HCl, pH 8.0 and 20% sucrose), incubated for 15 min at 22°C and centrifuged for 20 min at 10000 g and 4°C. The following purification steps were carried out at 4°C. The supernatant, containing the periplasmic proteins, was separated and centrifuged for 10 min at 20000 g. The crude extract was filtrated with a 0.45 µm pore-size filter membrane. Prior to immobilised metal affinity chromatography (IMAC) Imidazol (pH 8.0) and NaCl were added to concentrations of 5 mM and 250 mM, respectively. The sample was applied to a 5 ml HisTrap FF crude column (GE Healthcare) at 3 ml/min. The column was washed with IMAC-buffer (100 mM Tris/HCl, pH 8.0, 500 mM NaCl) supplemented with 15 mM imidazole at 1 ml/min. The nanobody was eluted from the column with IMAC-buffer supplemented with 500 mM imidazole at 1 ml/min. The fractions were analysed by SDS-PAGE, pooled and concentrated in a 3000 kDa cut-off AMICON ultrafiltration device (Merck-Millipore) to 10 mg/ml for gel permeation chromatography (GPC). GPC was performed in 10 mM Hepes, pH 7.5, 100 mM NaCl and 1mM $CaCl_2$ on a Superdex 75 100/300 GL column (GE Healthcare) at 0.5 ml/min. The fractions were analysed by SDS-PAGE, pooled and concentrated for crystallisation of Nb14, purification of the furin-Nb14 complex or inhibition/binding studies.

Human furin was expressed and purified as described previously [2]. In short, furin WT and the furin Thr562Arg (synthesised and cloned by GeneArt), both comprising the catalytic and the P-domain, were expressed in human embryonic kidney cells and purified by IMAC and immobilised inhibitor affinity chromatography. For complex formation with Nb14, furin was concentrated to 2.5 mg/ml (approx. 50µM) prior to GPC and treated by stepwise addition of dec-RVKR-CMK (Bachem) until the activity of the sample decreased below 1 %. Nb14 was added to the inhibited furin sample at twofold molar excess. The sample was concentrated in a 10000 kDa cut-off AMICON ultrafiltration device (Merck-Millipore) to 5 mg/ml and subjected to the GPC column (Superdex 200 100/300 GL; GE Healthcare) in 10 mM Hepes, pH 7.5, 100 mM NaCl and 2 mM $CaCl_2$ at 0.5 ml/min. Co-elution of the binding partners was analysed by SDS-PAGE. The fractions were pooled and concentrated (Vivaspin 500, 10000 kDa cut-off; Satorius) for crystallisation.

For binding and inhibition studies with recombinant factor X the buffer was exchanged after the immobilised inhibitor affinity chromatography step to 10 mM Hepes, pH 7.5, 100 mM NaCl and 1 mM $CaCl_2$.

Recombinant factor X^{S195A} was expressed and purified as described previously [3].

Crystallisation and structure determination.

Crystals of isolated Nb14 were grown in 96-well round bottom CrystalQuick plates (Greiner Bio One) applying a controlled dry-out procedure. Crystallisation drops (200 nl) of protein solution (10 mg/ml) were pipetted without any reservoir solution using a cartesian robot (Zinsser Analytic). The plates were sealed with ClearSeal film (Hampton research). Rod-shaped crystals grew after several days and were stable for several weeks. For mounting of the crystals 1 µl of stabilisation solution (1.93 M NaCl, 0.2 M Hepes, pH 7.5, 66 mM $CaCl_2$) was added to the drop. For flash cooling in liquid nitrogen the crystals were transferred to stabilisation solution supplemented with 25% glycerol. Diffraction data collection of single crystals at 100 K was performed at BL 14.1 of BESSY-II, Helmholtz-Zentrum Berlin (HZB) [4] (Table S1). The data were processed in XDS [5] (v.11/2013), the XDSAPP interface [6] (v.1.0) and with the CCP4-suite [7] (CCP4 v.6.3.0, CCP4 interface v.2.2.0). The structure was solved by molecular replacement (MR) in PHASER [8] using a camelid antibody fragment (PDB-ID: 1OP9, chain a, [9]) as search model, which was modified according to the amino acid sequence of Nb14. The model was built in COOT [10] (v.0.6.2) and refined in PHENIX [11] (v.1.9-1692) (Table S1).

Crystals of furin:RVKR-CMK:Nb14 complex grew in sitting drops prepared with equal volumes of protein (~9 mg/ml) and reservoir solution (0.1 M sodium acetate, pH 5.6, 16-18% PEG 3350). Heavily clustered crystals of needle-like shape grew after three weeks. Diffraction data were measured at 100 K with flash-cooled crystals by supplementation of the reservoir solution with 15% ethylene glycol. Initial data collection with a whole needle cluster was performed at BL 14.1 of BESSY-II (Table S1, Fig. S1a) [4]. Although invisible in the diffraction, image indexing with XDS [5] (v.11/2014) revealed a dominant crystal lattice belonging to the orthorhombic crystal system. The data were integrated and scaled with XDS [5] (v.11/2014) and used to solve the structure by MR in PHASER [8]. The refined structure of isolated Nb14 and the structure of human furin (PDB-ID 4RYD, [12]) were used as models in consecutive MR runs. Two copies of the protein-protein complex were identified in the asymmetric unit.

Needle clusters were also manipulated to obtain single crystals. Multiple fragments of plate-like and needle-like shape were mounted in elliptical loops (Molecular Dimensions). The crystals were flash-cooled as described for the needle clusters and measured at the micro-focus beamline P14, EMBL Hamburg (Table 1, Fig. S1b) with a 5x6 µm (FWHM) X-ray beam and a photon flux of $1x10^{12}$ photon/second. X-ray diffraction based sample centring was used to identify the start and end points of the helical data collection trajectory. 1500 diffraction images were recorded in 60 seconds using PILATUS2 6MF detector (DECTRIS Ltd, Baden, Switzerland), while the crystal was continuously rotated by 180° and translated by ~120 µm. The data were processed with XDS [5] (v.11/2014). The initial replacement solution was transferred to the high resolution data set. The complex structure was built and refined at 2.0 Å using COOT [10] (v.0.6.2) and PHENIX [11] (v.1.9-1692) (Table S1). Refinement parameters of the covalently bound decanoyl-Arg-Val-Lys-Arg-CMK peptide inhibitor were generated with PRODRG [13]. For furin two glycosylation sites were observed showing a clear electron density of the initial N-acetyl-glucosamine residue attached to Asn387 and Asn440. The refinement parameters were derived from the CCP4-monomer library as included in PHENIX [11] (v.1.9-1692) and applied using the apply_cif_link command as suggested in the PHENIX documentation v. 1.4. PHENIX [11] (v.1.9-1692) was used for calculation of simulated annealing composite-omit electron density maps (omitted region comprised 2.5% of the model). Structure alignments and molecular graphics were always generated with PYMOL (http://www.pymol.org).

For modelling of the furin:factor X:Nb14 complex, the structure of factor Xa (catalytic domain of factor X; PDB-ID: 2GD4; [14]) was aligned to the inhibitor peptide as observed in the RVKR-CMK:Nb14 complex. The catalytic domain of factor X was moved as a rigid body, varying initially only the relative conformation of the amino acids between Thr178 and Arg181 and later also of the peptide stretch Cys174 to Glu180.

Activity assays

Specific activity was determined as described previously [2]. Measurements were performed at 37°C in 100mM Hepes, 5mM CaCl, 0.5% TritonX100, pH7.0. The increase in fluorescence over 20 min by hydrolysis of pGlu-Arg-Thr-Lys-Arg-AMC (0.2 mM) was quantified with a FP-6500 spectro-fluorometer (Jasco, Excitation 380 nM, Emission 460 nM). Activity assays were performed as triplicates.

Sequence alignment

Sequences of furin-like human proprotein convertase isoforms were obtained from UNIPROT [15]: furin (P09958), PC1 (P29120), PC2 (P16519), PC4 (Q6UW60), PC5 (Q92824), PC7 (Q16549), PACE4 (P29122). The EMBL-EBI [16] CLUSTAL OMEGA web server [17] was used to create the multiple sequence alignment.

Supplementary figures

Figure S1

Crystals of the furin:RVKR-CMK:Nb14 complex, which were used for data collection. (a) Needle clusters were mounted in a nylon loop for data collection at BL14.1 of the Helmholtz-Zentrum Berlin (HZB) (Table S1). The approximate size of the X-ray beam is shown by the red oval. (b) Crystal fragments with approx. sizes of 5x5x150µm were mounted in an elliptical loop for helical data collection at beamline P14 at the European Molecular Biology Laboratory (EMBL) Hamburg (Table 1). The approximate size of the X-ray beam is shown by the blue rectangle.

Figure S2

Interaction interface of furin and Nb14. Furin and Nb14 are rotated by 45° in relation to Fig. 1 in opposite directions and the location of the interface region is marked in red. (a) Nb14 is represented as a cartoon (blue) with the central disulphide bond highlighted as spheres. (b) Furin is shown as a surface representation. The catalytic domain and the P-domain are coloured in yellow and brown, respectively. The RVKR-CMK inhibitor is shown as a ball and stick model with magenta carbons.

Figure S3

Overall structure of furin and Nb14. The protein backbone is always given as a cartoon representation. (a-b) Structural comparison of furin as observed in the complex with Nb14 (colouring according to Fig. 1) and antibody free furin (PDB-ID 4RYD[12], grey). The active side residues are shown as a stick model (cyan). The covalently bound RVKR-CMK inhibitor is shown as a ball and stick model. Calcium (green) and sodium (purple) ions are shown as spheres. (a) Furin is shown in standard orientation. (b) Furin rotated by 90° around the X-axis compared to A. The N-acetyl-glucosamine (GlcNac) residues of the glycosylation sites are shown as a stick model (grey carbons). (c) Structural comparison of Nb14 as observed in the complex with furin (blue) and isolated Nb14 (orange).

Figure S4

Modelling of the furin-factor X enzyme-substrate complex. The stereo panels show the furin:Nb14 complex as a surface representation. The catalytic domain of furin is coloured in yellow, the P-domain of furin is brown and Nb14 is blue. The catalytic domain of factor X (cartoon representation) was linked to the P1-P4 tetrapeptide (stick representation, magenta) of the co-crystallised RVKR-CMK inhibitor. (a) One conformer that fits to the furin:Nb14 complex is coloured in magenta. (b) The conformers that do not fit to the furin:Nb14 complex are given in grey.

Figure S5

Sequence-alignment of human furin-like proprotein convertase isoforms. Identical amino acids are shown in white on a black background. Catalytic residues are coloured in cyan. Amino acids of the interaction interface of furin and Nb14 are coloured in red. Thr562 is shown in white on a red background.

Supplementary tables

Table S1 **Data collection statistics**

	Furin+Nb14[c]	Furin+Nb14	Nb14
Beamline	BESSY BL14.1	P14, Petra-III, EMBL c/o DESY	BESSY BL14.1
Wavelength (Å)	0.918	0.9763	0.918
Unit cell parameters	$P2_12_12$	$P2_12_12$	$I4_122$
a (Å), b (Å), c (Å)	169.4, 50.0, 144.2	169.8, 50.0, 144.2	91.4, 91.4, 211,3
Resolution range [a] (Å)	48.1-2.9 (3.07-2.90)	66.4-2.0 (2.12-2.00)	35.4-2.3 (2.40-2.27)
R_{symm} [a] (%)	24.4 (56.9)	13.2 (60.8)	11.0 (70.4)
I/sigI [a]	6.5 (2.6)	12.0 (3.3)	11.9 (3.0)
Completeness [a] (%)	94.7 (96.2)	98.8 (97.1)	99.7 (99.4)
Observations: total / unique	122,346 / 26,618	560,939 / 83,341	139,425 / 21,253
**Refinement statistics**			
Rwork/Rfree		16.3/19.7	19.0/21.6
Non-hydrogen atoms		9,905	1,925
Protein/Inhibitor/Other		8,972/100/833	1,792/133
B-factors (Å^2):			
Overall/Wilson plot		23.4/26.4	30.3/35.0
Furin/Nb14/Inhibitor/Other		19.3/37.8/19.0/28.7	-/29.9/-/36.5
RMSD bond length (Å)		0.007	0.010
RMSD bonded B-factors (Å^2)		3.5	3.9

[a] Highest resolution shell is given in parentheses.

[b] $R_{symm}=\Sigma_h\Sigma_l|I_{hl}-\langle I_h\rangle|/ \Sigma_h\Sigma_l|\langle I_h\rangle|$

[c] Initial dataset of the mounted crystal bundle shown in Fig. S1A.

Supplementary references

1. *Zhu J, et al. Generation and characterization of non-competitive furin-inhibiting nanobodies. Biochem J **448**, 73-82 (2012).*

2. *Dahms SO, Hardes K, Becker GL, Steinmetzer T, Brandstetter H, Than ME. X-ray Structures of Human Furin in Complex with Competitive Inhibitors. ACS Chem Biol **9**, 1113-1118 (2014).*

3. *Sichler K, Kopetzki E, Huber R, Bode W, Hopfner KP, Brandstetter H. Physiological fIXa activation involves a cooperative conformational rearrangement of the 99-loop. J Biol Chem **278**, 4121-4126 (2003).*

4. *Mueller U, et al. Facilities for macromolecular crystallography at the Helmholtz-Zentrum Berlin. Journal of synchrotron radiation **19**, 442-449 (2012).*

5. *Kabsch W. Xds. Acta Crystallogr D Biol Crystallogr **66**, 125-132 (2010).*

6. *Krug M, Weiss MS, Heinemann U, Mueller U. XDSAPP: a graphical user interface for the convenient processing of diffraction data using XDS. J Appl Cryst **45**, 568-572 (2012).*

7. *Winn MD, et al. Overview of the CCP4 suite and current developments. Acta Crystallogr D Biol Crystallogr **67**, 235-242 (2011).*

8. *McCoy AJ, Grosse-Kunstleve RW, Adams PD, Winn MD, Storoni LC, Read RJ. Phaser crystallographic software. Journal of applied crystallography **40**, 658-674 (2007).*

9. *Dumoulin M, et al. A camelid antibody fragment inhibits the formation of amyloid fibrils by human lysozyme. Nature **424**, 783-788 (2003).*

10. *Emsley P, Lohkamp B, Scott WG, Cowtan K. Features and development of Coot. Acta Crystallogr D Biol Crystallogr **66**, 486-501 (2010).*

11. *Adams PD, et al. PHENIX: a comprehensive Python-based system for macromolecular structure solution. Acta Crystallogr D Biol Crystallogr **66**, 213-221 (2010).*

12. *Hardes K, et al. Novel Furin Inhibitors with Potent Anti-infectious Activity. ChemMedChem, (2015).*

13. *Schuttelkopf AW, van Aalten DM. PRODRG: a tool for high-throughput crystallography of protein-ligand complexes. Acta Crystallogr D Biol Crystallogr **60**, 1355-1363 (2004).*

14. *Johnson DJ, Li W, Adams TE, Huntington JA. Antithrombin-S195A factor Xa-heparin structure reveals the allosteric mechanism of antithrombin activation. The EMBO journal* ***25****, 2029-2037 (2006).*

15. *UniProt: a hub for protein information. Nucleic acids research* ***43****, D204-212 (2015).*

16. *Li W, et al. The EMBL-EBI bioinformatics web and programmatic tools framework. Nucleic acids research* ***43****, W580-584 (2015).*

17. *Sievers F, et al. Fast, scalable generation of high-quality protein multiple sequence alignments using Clustal Omega. Molecular systems biology* ***7****, 539 (2011).*

Dahms et al. 2016, Fig. S1

(a)

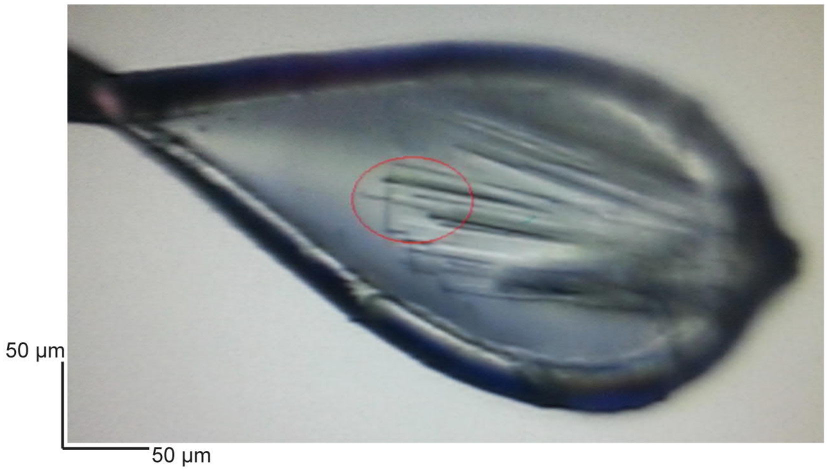

(b)

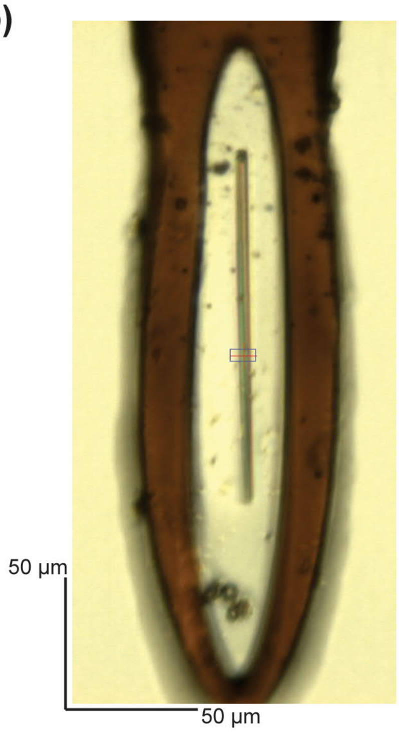

Dahms et al. 2016, Fig. S2

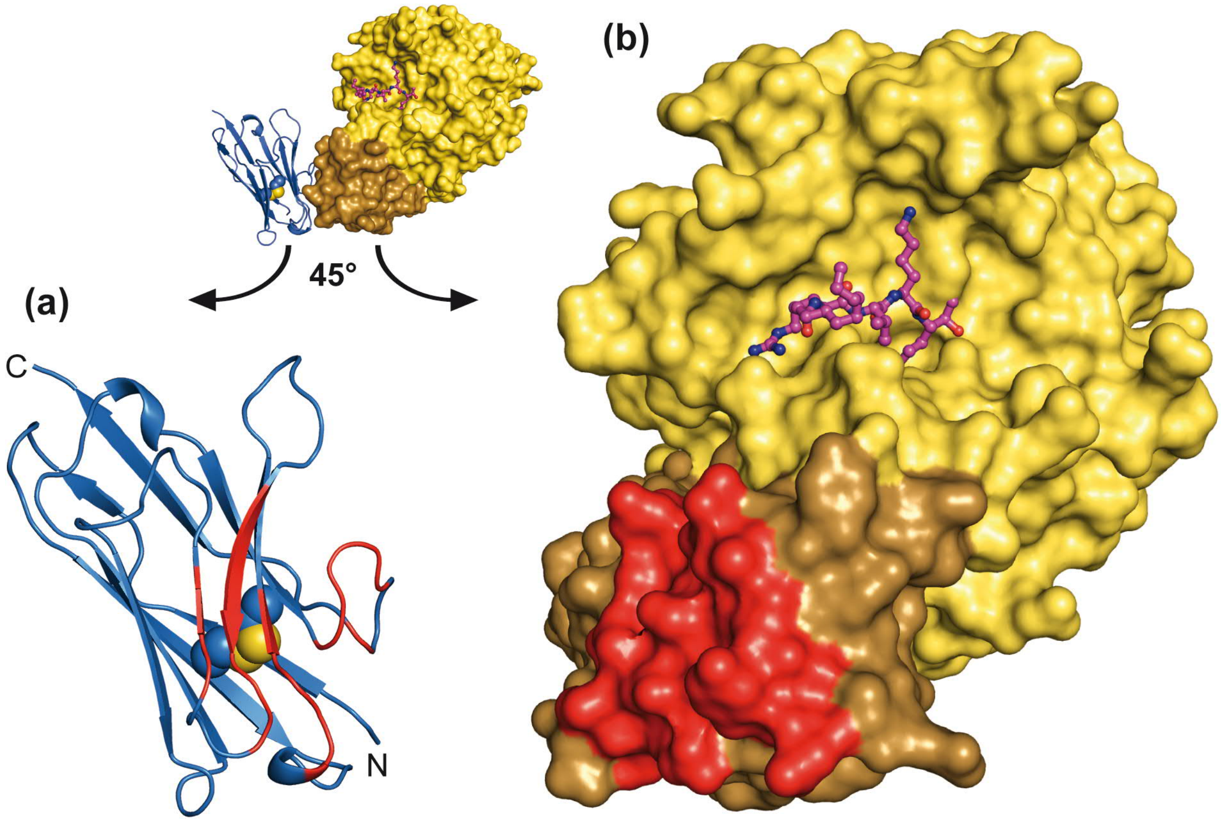

Dahms et al. 2016, Fig. S3

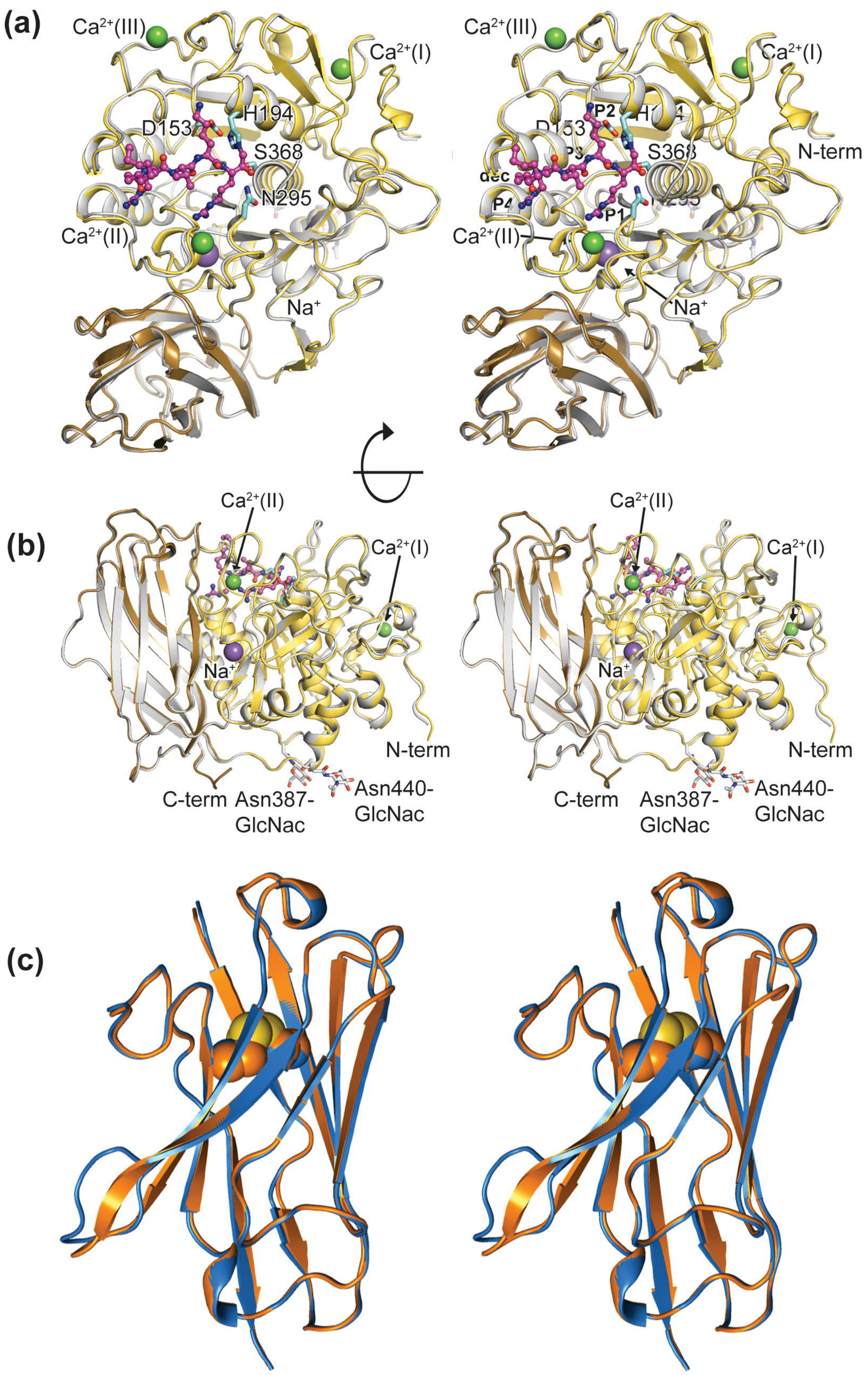

Dahms et al. 2016, Fig. S4

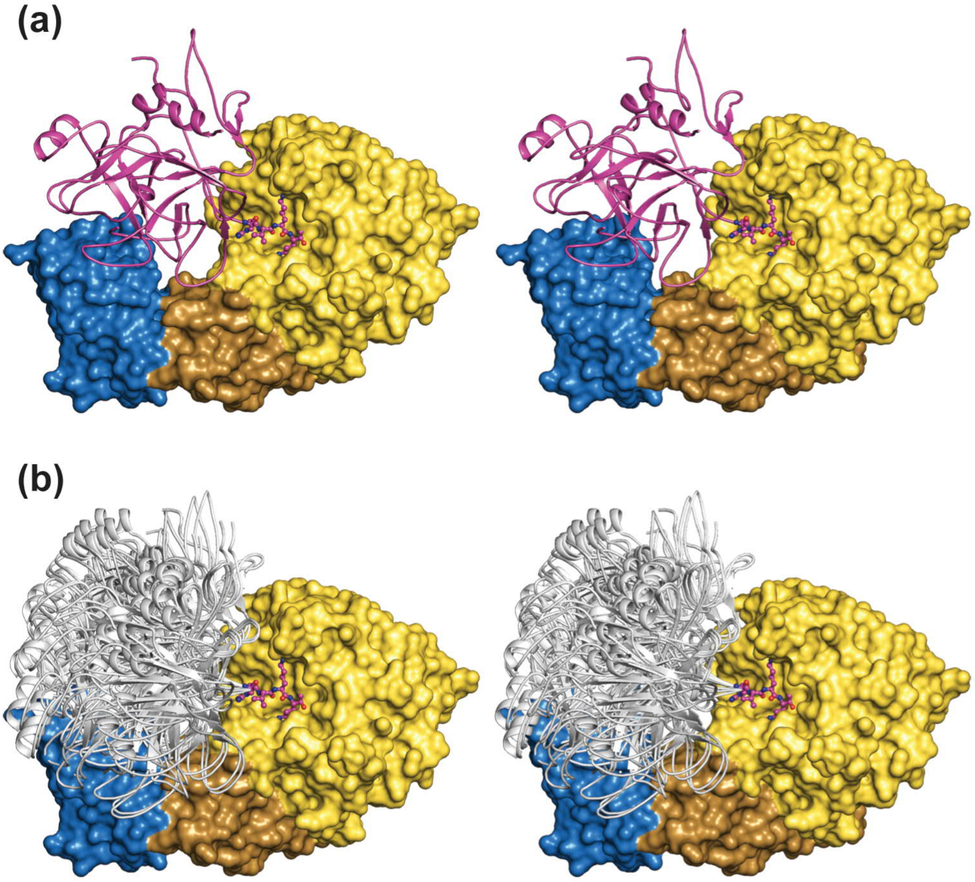

Dahms et al. 2016, Fig. S5

```
Domain:          catalytic domain
furin    108     ------DVYQEPTDPKFPQQWYLSG------VTQRDLNVKAAWAQGYTCHGIVVSILDDG
PC1      111     -SALRDSALNLFNDPMWNQQWYLQDTRMTAALPKLDLHVIPVWQKGITGKGVVITVLDDG
PC2      110     GYRDINEIDINMNDPLFTKQWYLINTGQADGTPGLDLNVAEAWELGYTGKGVTIGIMDDG
PC4      114     -------SVVVPTDPWFSKQWYMNS------EAQPDLSILQAWSQGLSGQGIVVSVLDDG
PC5      115     DYDFSRAQSTYFNDPKWPSMWYMHCSDNT-HPCQSDMNIEGAWKRGYTGKNIVVTILDDG
PC7      142     --------SVHFNDPKYPQQWHLNNRR----SPGRDINVTGVWERNVTGRGVTVVVVDDG
PACE4    150     -QVRSDPQALYFNDPIWSNMWYLHCGDKN-SRCRSEMNVQAAWKRGYTGKNVVVTILDDG

Domain:          catalytic domain
furin    156     IEKNHPDLAGNYDPGASFDVNDQDPDPQPRYTQMNDNRHGTRCAGEVAAVANNGVCGVGV
PC1      170     LEWNHTDIYANYDPEASYDFNDNDHDPFPRYDPTNENKHGTRCAGEIAMQANNHKCGVGV
PC2      170     IDYLHPDLASNYNAEASYDFSSNDPYPYPRYTDDWFNSHGTRCAGEVSAAANNNICGVGV
PC4      161     IEKDHPDLWANYDPLASYDFNDYDPDPQPRYTPSKENRHGTRCAGEVAAMANNGFCGVGV
PC5      174     IERTHPDLMQNYDALASCDVNGNDLDPMPRYDASNENKHGTRCAGEVAAAANNSHCTVGI
PC7      190     VEHTIQDIAPNYSPEGSYDLNSNDPDPMPHPDVENGNHHGTRCAGEIAAVPNNSFCAVGV
PACE4    208     IERNHPDLAPNYDSYASYDVNGNDYDPSPRYDASNENKHGTRCAGEVAASANNSYCIVGI

Domain:          catalytic domain
furin    216     AYNARIGGVRMLDGE-VTDAVEARSLGLNPNHIHIYSASWGPEDDGKTVDGPARLAEEAF
PC1      230     AYNSKVGGIRMLDGI-VTDAIEASSIGFNPGHVDIYSASWGPNDDGKTVEGPGRLAQKAF
PC2      230     AYNSKVAGIRMLDQPFMTDIIEASSISHMPQLIDIYSASWGPTDNGKTVDGPRELTLQAM
PC4      221     AFNARIGGVRMLDGT-ITDVIEAQSLSLQPQHIHIYSASWGPEDDGRTVDGPGILTREAF
PC5      234     AFNAKIGGVRMLDGD-VTDMVEAKSVSFNPQHVHIYSASWGPDDDGKTVDGPAPLTRQAF
PC7      250     AYGSRIAGIRVLDGP-LTDSMEAVAFNKHYQINDIYSCSWGPDDDGKTVDGPHQLGKAAL
PACE4    268     AYNAKIGGIRMLDGD-VTDVVEAKSLGIRPNYIDIYSASWGPDDDGKTVDGPGRLAKQAF

Domain:          catalytic domain
furin    275     FRGVSQGRGGLGSIFVWASGNGGREHDSCNCDGYTNSIYTLSISSATQFGNVPWYSEACS
PC1      289     EYGVKQGRQGKGSIFVWASGNGGRQGDNCDCDGYTDSIYTISISSASQQGLSPWYAEKCS
PC2      290     ADGVNKGRGGKGSIYVWASGDGG-SYDDCNCDGYASSMWTISINSAINDGRTALYDESCS
PC4      280     RRGVTKGRGGLGTLFIWASGNGGLHYDNCNCDGYTNSIHTLSVGSTTQQGRVPWYSEACA
PC5      293     ENGVRMGRRGLGSVFVWASGNGGRSKDHCSCDGYTNSIYTISISSTAESGKKPWYLEECS
PC7      309     QHGVIAGRQGFGSIFVVASGNGGQHNDNCNYDGYANSIYTVTIGAVDEEGRMPFYAEECA
PACE4    327     EYGIKKGRQGLGSIFVWASGNGGREGDYCSCDGYTNSIYTISVSSATENGYKPWYLEECA

Domain:          catalytic domain
furin    335     STLATTYSSGNQNE--KQIVTTDL----RQKCTESHTGTSASAPLAAGIIALTLEANKNL
PC1      349     STLATSYSSGDYTD--QRITSADL----HNDCTETHTGTSASAPLAAGIFALALEANPNL
PC2      349     STLASTFSNGRKRNPEAGVATTDL----YGNCTLRHSGTSAAAPEAAGVFALALEANLGL
PC4      340     STLTTTYSSGVATD--PQIVTTDL----HHGCTDQHTGTSASAPLAAGMIALALEANPFL
PC5      353     STLATTYSSGESYD--KKIITTDL----RQRCTDNHTGTSASAPMAAGIIALALEANPFL
PC7      369     SMLAVTFSGGDKML--RSIVTTDWDLQKGTGCTEGHTGTSAAAPLAAGMIALMLQVRPCL
PACE4    387     STLATTYSSGAFYE--RKIVTTDL----RQRCTDGHTGTSVSAPMVAGIIALALEANSQL

Domain:          catalytic domain
furin    389     TWRDMQHLVVQTSKPAHL--NANDWATNGVGRKVSHSYGYGLLDAGAMVALA--QNWTTV
PC1      403     TWRDMQHLVVWTSEYDPLA-NNPGWKKNGAGLMVNSRFGFGLLNAKALVDLADPRTWRSV
PC2      405     TWRDMQHLTVLTSKRNQLHDEVHQWRRNGVGLEFNHLFGYGVLDAGAMVKMA--KDWKTV
PC4      394     TWRDMQHLVVRASKPAHL--QAEDWRTNGVGRQVSHHYGYGLLDAGLLVDTA--RTWLPT
PC5      407     TWRDVQHVIVRTSRAGHL--NANDWKTNAAGFKVSHLYGFGLMDAEAMVMEA--EKWTTV
PC7      427     TWRDVQHIIVFTATRYE--DRRAEWVTNEAGFSHSHQHGFGLLNAWRLVNAA--KIWTSV
PACE4    441     TWRDVQHLLVKTSRPAHL--KASDWKVNGAGHKVSHFYGFGLVDAEALVVEA--KKWTAV

Domain:          P-domain
furin    445     APQRKCIIDI-LTEPKDIGKRLEVRKT--VTACLGEP-NHITRLEHAQARLTLSYNRRGD
PC1      462     PEKKECVVKDNDFEPRALKANGEVIIEIPTRACEGQE-NAIKSLEHVQFEATIEYSRRGD
PC2      463     PERFHCVGGS-VQDPEKIPSTGKLVLTLTTDACEGKE-NFVRYLEHVQAVITVNATRRGD
PC4      450     QPQRKCAVRV-QSRPTPILPLIYIREN--VSACAGLH-NSIRSLEHVQAQLTLSYSRRGD
PC5      463     PRQHVCVEST-DRQIKTIRPNSAVRSIYKASGCSDNPNRHVNYLEHVVVRITITHPRRGD
PC7      483     PYLASYVSPV-LKENKAIPQSPRSLEVLWNVSRMDLEMSGLKTLEHVAVTVSITHPRRGS
PACE4    497     PSQHMCVAAS-DKRPRSIPLVQVLRTTALTSACAEHSDQRVVYLEHVVVRTSISHPRRGD

Domain:          P-domain
furin    501     LAIHLVSPMGTRSTLLAARPHD-YSADGFNDWAFMTTHSWDEDPSGEWVLEIENTSE---
PC1      521     LHVTLTSAAGTSTVLLAERERD-TSPNGFKNWDFMSVHTWGENPIGTWTLRITDMSGRI-
PC2      521     LNINMTSPMGTKSILLSRRPRDDDSKVGFDKWPFMTTHTWGEDARGTWTLELGFVGS---
PC4      506     LEISLTSPMGTRSTLVAIRPLD-VSTEGYNNWVFMSTHFWDENPQGVWTLGLENKGY---
PC5      522     LAIYLTSPSGTRSQLLANRLFD-HSMEGFKNWEFMTIHCWGERAAGDWVLEVYDTPSQLR
PC7      542     LELKLFCPSGMMSLIGAPRSMD-SDPNGFNDWTFSTVRCWGERARGTYRLVIRDVGDE--
PACE4    556     LQIYLVSPSGTKSQLLAKRLLD-LSNEGFTNWEFMTVHCWGEKAEGQWTLEIQDLPSQVR

Domain:          P-domain
furin    557     -ANNYGTLTKFTLVLYGTA
PC1      579     --QNEGRIVNWKLILHGTS
PC2      578     -APQKGVLKEWTLMLHGTQ
PC4      562     -YFNTGTLYRYTLLLYGTA
PC5      581     NFKTPGKLKEWSLVLYGTS
PC7      599     -SFQVGILRQWQLTLYGSV
PACE4    615     NPEKQGKLKEWSLILYGTA
```
